# 3-[2-Chloro-4-(trifluoro­meth­yl)phen­oxy]benzoic acid

**DOI:** 10.1107/S1600536811043479

**Published:** 2011-10-29

**Authors:** Yan-Ju Liu, Jie Liu

**Affiliations:** a Pharmacy College, Henan University of Traditional Chinese Medicine, Zhengzhou 450008, People’s Republic of China; b Department of Urology, Henan Provincial People’s Hospital, Zhengzhou 450003, People’s Republic of China

## Abstract

The asymmetric unit of the title compound, C_14_H_8_ClF_3_O_3_, comprises two independent mol­ecules. The rings in each molecule are connected together *via* O—H⋯O hydrogen bonds to form classical hydrogen-bonded carb­oxy­lic acid dimers. The dihedral angles between the benzene rings are 80.7 (1) and 68.7 (1)°.

## Related literature

For background on applications of the title compound, see: Brown *et al.* (1997 ▶). For the synthesis of the title compound, see: Johnson (1977 ▶). For bond-length data, see: Allen *et al.* (1987 ▶).
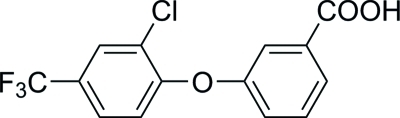


## Experimental

### 

#### Crystal data

C_14_H_8_ClF_3_O_3_
*M* *_r_* = 316.66
Triclinic, 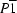

*a* = 7.3390 (15) Å
*b* = 7.6880 (15) Å
*c* = 24.113 (5) Å
α = 90.54 (3)°
β = 92.18 (3)°
γ = 94.23 (3)°
*V* = 1355.7 (5) Å^3^
*Z* = 4
Mo *K*α radiation
μ = 0.32 mm^−1^
*T* = 293 K
0.30 × 0.30 × 0.10 mm

#### Data collection

Enraf–Nonius CAD-4 diffractometer
Absorption correction: ψ scan (North *et al.*, 1968 ▶) *T*_min_ = 0.909, *T*_max_ = 0.968
5407 measured reflections
4984 independent reflections
2318 reflections with *I* > 2σ(*I*)
*R*_int_ = 0.044
3 standard reflections every 200 reflections  intensity decay: 1%

#### Refinement

*R*[*F* ^2^ > 2σ(*F* ^2^)] = 0.068
*wR*(*F* ^2^) = 0.167
*S* = 1.00
4984 reflections
379 parameters
H-atom parameters constrained
Δρ_max_ = 0.30 e Å^−3^
Δρ_min_ = −0.23 e Å^−3^

### 

Data collection: *CAD-4 Software* (Enraf–Nonius, 1985 ▶); cell refinement: *CAD-4 Software*; data reduction: *XCAD4* (Harms & Wocadlo, 1995 ▶); program(s) used to solve structure: *SHELXS97* (Sheldrick, 2008 ▶); program(s) used to refine structure: *SHELXL97* (Sheldrick, 2008 ▶); molecular graphics: *SHELXTL* (Sheldrick, 2008 ▶); software used to prepare material for publication: *SHELXTL*.

## Supplementary Material

Crystal structure: contains datablock(s) I, global. DOI: 10.1107/S1600536811043479/vm2129sup1.cif

Structure factors: contains datablock(s) I. DOI: 10.1107/S1600536811043479/vm2129Isup2.hkl

Supplementary material file. DOI: 10.1107/S1600536811043479/vm2129Isup3.cml

Additional supplementary materials:  crystallographic information; 3D view; checkCIF report

## Figures and Tables

**Table 1:** Hydrogen-bond geometry (Å, °)

*D*—H⋯*A*	*D*—H	H⋯*A*	*D*⋯*A*	*D*—H⋯*A*
O2—H2*B*⋯O6	0.82	1.79	2.599 (4)	168
O5—H5*B*⋯O3	0.82	1.82	2.629 (4)	170

**Fig. 1.**
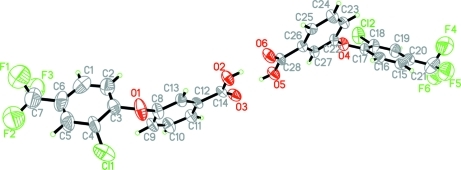
The molecular structure of (I), with the atom-numbering scheme. Displacement ellipsoids are drawn at the 50% probability level.

**Fig. 2.**
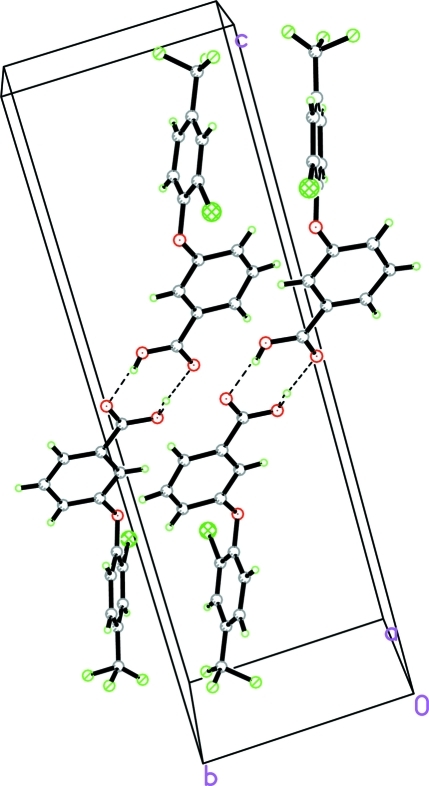
A packing diagram of (I). Hydrogen bonds are shown as dashed lines.

## supplementary crystallographic information

### Comment

The title compound, 3-[2-chloro-4-(trifluoromethyl)phenoxy]benzoic acid is an important intermediate, which can be utilized to synthesize acifluorfen (Brown *et al.*, 1997). Here we report the crystal structure of the title compound Fig. 1). The asymmetric unit contains two molecules with a similar conformation (rms deviation fitting 21 non-H atoms: 0.400 Å) .

Intermolecular O—H···O hydrogen bonds (Table 1, Fig. 2) result in the formation of carboxylic acid dimers. The bond lengths and angles are within normal ranges (Allen *et al.*, 1987). The dihedral angles between the rings A(C1—C6), B(C8—C13), C(C15—C20), D(C22—C27) are: A/B = 80.7 (1)°, C/D = 68.7 (1)°. The O atoms O1 and O4 lie in the benzene ring planes A and B, and C and D, respectively.

### Experimental

The title compound, (I) was prepared by the method of the Ullmann condensation reaction (Johnson, 1977). The crystals were obtained by dissolving (I) (0.2 g, 0.6 mmol) in ethanol (25 ml) and evaporating the solvent slowly at room temperature for about 5 d.

### Refinement

H atoms were positioned geometrically and refined as riding groups, with O—H = 0.82 and C—H = 0.93 Å for aromatic H, and with *U*_iso_(H) = *xU*_eq_(C), where *x* = 1.2 for aromatic H, and *x* = 1.5 for other H.

### Crystal data

**Table:**

C_14_H_8_ClF_3_O_3_	*Z* = 4
*M**_r_* = 316.66	*F*(000) = 640
Triclinic, *P*1	*D*_x_ = 1.551 Mg m^−^^3^
Hall symbol: -P 1	Mo *K*α radiation, λ = 0.71073 Å
*a* = 7.3390 (15) Å	Cell parameters from 25 reflections
*b* = 7.6880 (15) Å	θ = 9–12°
*c* = 24.113 (5) Å	µ = 0.32 mm^−^^1^
α = 90.54 (3)°	*T* = 293 K
β = 92.18 (3)°	Block, colourless
γ = 94.23 (3)°	0.30 × 0.30 × 0.10 mm
*V* = 1355.7 (5) Å^3^	

### Data collection

**Table:**

Enraf–Nonius CAD-4 diffractometer	2318 reflections with *I* > 2σ(*I*)
Radiation source: fine-focus sealed tube	*R*_int_ = 0.044
graphite	θ_max_ = 25.4°, θ_min_ = 1.7°
ω/2θ scans	*h* = 0→8
Absorption correction: ψ scan (North *et al.*, 1968)	*k* = −9→9
*T*_min_ = 0.909, *T*_max_ = 0.968	*l* = −29→29
5407 measured reflections	3 standard reflections every 200 reflections
4984 independent reflections	intensity decay: 1%

### Refinement

**Table:**

Refinement on *F*^2^	Primary atom site location: structure-invariant direct methods
Least-squares matrix: full	Secondary atom site location: difference Fourier map
*R*[*F*^2^ > 2σ(*F*^2^)] = 0.068	Hydrogen site location: inferred from neighbouring sites
*wR*(*F*^2^) = 0.167	H-atom parameters constrained
*S* = 1.00	*w* = 1/[σ^2^(*F*_o_^2^) + (0.040*P*)^2^ + 1.5*P*] where *P* = (*F*_o_^2^ + 2*F*_c_^2^)/3
4984 reflections	(Δ/σ)_max_ < 0.001
379 parameters	Δρ_max_ = 0.30 e Å^−^^3^
0 restraints	Δρ_min_ = −0.23 e Å^−^^3^

### Special details

**Table:**

Geometry. All e.s.d.'s (except the e.s.d. in the dihedral angle between two l.s. planes) are estimated using the full covariance matrix. The cell e.s.d.'s are taken into account individually in the estimation of e.s.d.'s in distances, angles and torsion angles; correlations between e.s.d.'s in cell parameters are only used when they are defined by crystal symmetry. An approximate (isotropic) treatment of cell e.s.d.'s is used for estimating e.s.d.'s involving l.s. planes.
Refinement. Refinement of *F*^2^ against ALL reflections. The weighted *R*-factor *wR* and goodness of fit *S* are based on *F*^2^, conventional *R*-factors *R* are based on *F*, with *F* set to zero for negative *F*^2^. The threshold expression of *F*^2^ > σ(*F*^2^) is used only for calculating *R*-factors(gt) *etc*. and is not relevant to the choice of reflections for refinement. *R*-factors based on *F*^2^ are statistically about twice as large as those based on *F*, and *R*- factors based on ALL data will be even larger.

### Fractional atomic coordinates and isotropic or equivalent isotropic displacement parameters (Å^2^)

**Table:**

	*x*	*y*	*z*	*U*_iso_*/*U*_eq_	
Cl1	0.9919 (2)	0.7043 (2)	0.23190 (8)	0.0893 (6)	
F1	0.5275 (7)	0.6798 (7)	0.0264 (2)	0.1418 (17)	
F2	0.7104 (7)	0.8940 (6)	0.0453 (2)	0.1387 (17)	
F3	0.3837 (7)	0.8748 (6)	0.05963 (17)	0.1223 (15)	
O1	0.6596 (5)	0.5437 (5)	0.27733 (15)	0.0721 (12)	
C1	0.4377 (10)	0.6640 (9)	0.1489 (3)	0.089 (2)	
H1A	0.3208	0.6606	0.1325	0.107*	
O2	0.3868 (4)	0.2435 (4)	0.43749 (13)	0.0508 (9)	
H2B	0.3410	0.1827	0.4618	0.076*	
C2	0.4616 (8)	0.5938 (8)	0.1999 (3)	0.0747 (18)	
H2A	0.3642	0.5362	0.2174	0.090*	
O3	0.2751 (4)	0.4507 (4)	0.48761 (13)	0.0530 (9)	
C3	0.6323 (7)	0.6100 (6)	0.2247 (2)	0.0524 (14)	
C4	0.7807 (8)	0.6875 (7)	0.1984 (2)	0.0606 (15)	
C5	0.7505 (11)	0.7526 (8)	0.1467 (3)	0.082 (2)	
H5A	0.8485	0.8071	0.1287	0.098*	
C6	0.5840 (12)	0.7410 (9)	0.1208 (3)	0.081 (2)	
C7	0.5508 (15)	0.8149 (13)	0.0650 (3)	0.104 (3)	
C8	0.5568 (6)	0.5995 (6)	0.3193 (2)	0.0487 (13)	
C9	0.5181 (6)	0.7782 (6)	0.3263 (2)	0.0515 (14)	
H9A	0.5595	0.8621	0.3013	0.062*	
C10	0.4214 (7)	0.8220 (7)	0.3695 (2)	0.0602 (15)	
H10A	0.3868	0.9357	0.3719	0.072*	
C11	0.3704 (6)	0.7070 (6)	0.4106 (2)	0.0474 (13)	
H11A	0.3118	0.7434	0.4417	0.057*	
C12	0.4109 (5)	0.5314 (6)	0.40349 (19)	0.0375 (11)	
C13	0.5042 (6)	0.4793 (6)	0.35821 (19)	0.0441 (12)	
H13A	0.5311	0.3636	0.3542	0.053*	
C14	0.3558 (6)	0.4010 (6)	0.4459 (2)	0.0391 (11)	
Cl2	−0.43627 (19)	−0.1013 (2)	0.78752 (7)	0.0802 (5)	
F4	−0.0534 (7)	−0.4636 (7)	0.94183 (19)	0.1401 (17)	
F5	0.1853 (7)	−0.3609 (6)	0.93988 (17)	0.1149 (14)	
F6	0.0134 (6)	−0.2118 (6)	0.9690 (2)	0.1243 (16)	
O4	−0.1400 (4)	−0.1045 (4)	0.71473 (13)	0.0473 (9)	
O5	0.1479 (4)	0.2192 (4)	0.55868 (13)	0.0543 (9)	
H5B	0.1906	0.2815	0.5342	0.082*	
O6	0.2627 (4)	0.0143 (4)	0.50788 (13)	0.0556 (10)	
C15	0.1275 (7)	−0.2544 (7)	0.8357 (2)	0.0587 (15)	
H15A	0.2448	−0.2851	0.8452	0.070*	
C16	0.0786 (6)	−0.2065 (7)	0.7814 (2)	0.0543 (14)	
H16A	0.1662	−0.2086	0.7546	0.065*	
C17	−0.0920 (6)	−0.1566 (6)	0.76583 (19)	0.0384 (11)	
C18	−0.2194 (6)	−0.1585 (6)	0.8058 (2)	0.0493 (13)	
C19	−0.1786 (8)	−0.2077 (7)	0.8604 (2)	0.0623 (15)	
H19A	−0.2678	−0.2089	0.8868	0.075*	
C20	−0.0074 (8)	−0.2536 (7)	0.8745 (2)	0.0587 (15)	
C21	0.0403 (11)	−0.3023 (13)	0.9317 (3)	0.084 (2)	
C22	−0.0435 (5)	−0.1560 (6)	0.66983 (17)	0.0336 (11)	
C23	−0.0255 (6)	−0.3304 (6)	0.66038 (19)	0.0444 (12)	
H23A	−0.0697	−0.4140	0.6851	0.053*	
C24	0.0622 (6)	−0.3792 (6)	0.61181 (19)	0.0458 (13)	
H24A	0.0742	−0.4963	0.6039	0.055*	
C25	0.1299 (6)	−0.2522 (6)	0.57632 (18)	0.0362 (11)	
H25A	0.1911	−0.2844	0.5452	0.043*	
C26	0.1077 (5)	−0.0778 (6)	0.58650 (18)	0.0339 (10)	
C27	0.0201 (6)	−0.0255 (6)	0.63470 (18)	0.0403 (11)	
H27A	0.0059	0.0914	0.6424	0.048*	
C28	0.1791 (6)	0.0550 (6)	0.54833 (18)	0.0380 (11)	

### Atomic displacement parameters (Å^2^)

**Table:**

	*U*^11^	*U*^22^	*U*^33^	*U*^12^	*U*^13^	*U*^23^
Cl1	0.0652 (11)	0.0732 (11)	0.1288 (16)	−0.0061 (8)	0.0134 (10)	0.0146 (10)
F1	0.153 (4)	0.148 (4)	0.124 (4)	0.007 (4)	−0.001 (3)	−0.003 (3)
F2	0.140 (4)	0.145 (4)	0.131 (4)	0.005 (3)	0.012 (3)	0.011 (3)
F3	0.128 (4)	0.129 (4)	0.111 (4)	0.019 (3)	0.001 (3)	0.006 (3)
O1	0.077 (3)	0.080 (3)	0.068 (3)	0.039 (2)	0.045 (2)	0.029 (2)
C1	0.094 (6)	0.097 (5)	0.075 (5)	0.016 (4)	−0.017 (4)	−0.014 (4)
O2	0.058 (2)	0.043 (2)	0.053 (2)	0.0089 (17)	0.0285 (17)	0.0066 (16)
C2	0.071 (5)	0.087 (5)	0.067 (4)	0.011 (4)	0.004 (4)	0.010 (4)
O3	0.058 (2)	0.047 (2)	0.055 (2)	−0.0042 (17)	0.0294 (18)	−0.0059 (17)
C3	0.052 (3)	0.050 (3)	0.057 (4)	0.011 (3)	0.014 (3)	0.009 (3)
C4	0.078 (4)	0.052 (3)	0.053 (4)	−0.001 (3)	0.031 (3)	0.010 (3)
C5	0.112 (6)	0.072 (4)	0.066 (5)	0.009 (4)	0.042 (4)	0.000 (4)
C6	0.115 (6)	0.082 (5)	0.050 (4)	0.031 (5)	0.022 (4)	−0.012 (4)
C7	0.128 (8)	0.112 (7)	0.074 (6)	0.012 (6)	0.006 (6)	0.005 (5)
C8	0.043 (3)	0.054 (3)	0.054 (3)	0.021 (3)	0.018 (3)	0.017 (3)
C9	0.052 (3)	0.047 (3)	0.056 (4)	−0.002 (3)	0.021 (3)	0.013 (3)
C10	0.054 (3)	0.053 (3)	0.077 (4)	0.022 (3)	0.013 (3)	0.024 (3)
C11	0.046 (3)	0.044 (3)	0.054 (3)	0.012 (2)	0.012 (2)	0.003 (2)
C12	0.022 (2)	0.043 (3)	0.047 (3)	−0.001 (2)	0.011 (2)	0.010 (2)
C13	0.034 (3)	0.055 (3)	0.045 (3)	0.004 (2)	0.012 (2)	0.009 (2)
C14	0.037 (3)	0.030 (3)	0.050 (3)	0.000 (2)	0.010 (2)	0.008 (2)
Cl2	0.0459 (9)	0.1133 (13)	0.0872 (12)	0.0282 (8)	0.0288 (8)	0.0293 (10)
F4	0.145 (4)	0.150 (5)	0.123 (4)	0.000 (4)	−0.007 (3)	0.013 (3)
F5	0.120 (4)	0.130 (4)	0.095 (3)	0.011 (3)	0.001 (3)	0.009 (3)
F6	0.140 (4)	0.129 (4)	0.104 (4)	0.010 (3)	0.004 (3)	0.004 (3)
O4	0.0397 (19)	0.056 (2)	0.049 (2)	0.0124 (16)	0.0194 (16)	0.0079 (17)
O5	0.073 (2)	0.042 (2)	0.050 (2)	0.0014 (18)	0.0302 (18)	−0.0007 (16)
O6	0.068 (2)	0.049 (2)	0.051 (2)	−0.0010 (18)	0.0369 (19)	0.0058 (17)
C15	0.050 (3)	0.066 (4)	0.058 (4)	−0.006 (3)	0.007 (3)	−0.002 (3)
C16	0.033 (3)	0.072 (4)	0.057 (4)	−0.006 (3)	0.005 (3)	−0.001 (3)
C17	0.027 (3)	0.050 (3)	0.039 (3)	0.005 (2)	0.009 (2)	0.010 (2)
C18	0.039 (3)	0.067 (4)	0.044 (3)	0.014 (3)	0.020 (2)	0.004 (3)
C19	0.069 (4)	0.077 (4)	0.042 (3)	0.005 (3)	0.016 (3)	0.008 (3)
C20	0.069 (4)	0.047 (3)	0.059 (4)	−0.001 (3)	−0.002 (3)	0.003 (3)
C21	0.065 (5)	0.139 (8)	0.051 (5)	0.010 (5)	0.012 (4)	−0.004 (5)
C22	0.022 (2)	0.046 (3)	0.034 (3)	0.006 (2)	0.012 (2)	0.012 (2)
C23	0.047 (3)	0.040 (3)	0.046 (3)	0.000 (2)	0.008 (2)	0.009 (2)
C24	0.045 (3)	0.040 (3)	0.055 (3)	0.013 (2)	0.017 (3)	0.006 (2)
C25	0.035 (3)	0.039 (3)	0.035 (3)	0.006 (2)	0.011 (2)	−0.006 (2)
C26	0.027 (2)	0.036 (3)	0.041 (3)	0.008 (2)	0.009 (2)	0.006 (2)
C27	0.040 (3)	0.043 (3)	0.039 (3)	0.009 (2)	0.005 (2)	0.009 (2)
C28	0.039 (3)	0.042 (3)	0.034 (3)	0.002 (2)	0.015 (2)	0.007 (2)

### Geometric parameters (Å, °)

**Table:**

Cl1—C4	1.717 (6)	Cl2—C18	1.724 (5)
F1—C7	1.385 (9)	F4—C21	1.401 (9)
F2—C7	1.382 (9)	F5—C21	1.196 (7)
F3—C7	1.344 (9)	F6—C21	1.163 (7)
O1—C8	1.369 (5)	O4—C17	1.342 (5)
O1—C3	1.385 (6)	O4—C22	1.387 (5)
C1—C2	1.356 (8)	O5—C28	1.323 (5)
C1—C6	1.390 (9)	O5—H5B	0.8200
C1—H1A	0.9300	O6—C28	1.222 (5)
O2—C14	1.264 (5)	C15—C20	1.389 (7)
O2—H2B	0.8200	C15—C16	1.403 (7)
C2—C3	1.365 (7)	C15—H15A	0.9300
C2—H2A	0.9300	C16—C17	1.376 (6)
O3—C14	1.257 (5)	C16—H16A	0.9300
C3—C4	1.381 (6)	C17—C18	1.368 (6)
C4—C5	1.362 (8)	C18—C19	1.400 (7)
C5—C6	1.349 (8)	C19—C20	1.362 (7)
C5—H5A	0.9300	C19—H19A	0.9300
C6—C7	1.483 (9)	C20—C21	1.468 (9)
C8—C13	1.370 (6)	C22—C23	1.375 (6)
C8—C9	1.433 (6)	C22—C27	1.385 (5)
C9—C10	1.337 (6)	C23—C24	1.418 (6)
C9—H9A	0.9300	C23—H23A	0.9300
C10—C11	1.378 (6)	C24—C25	1.381 (6)
C10—H10A	0.9300	C24—H24A	0.9300
C11—C12	1.413 (6)	C25—C26	1.384 (5)
C11—H11A	0.9300	C25—H25A	0.9300
C12—C13	1.383 (6)	C26—C27	1.418 (6)
C12—C14	1.485 (6)	C26—C28	1.462 (6)
C13—H13A	0.9300	C27—H27A	0.9300
			
C8—O1—C3	118.6 (4)	C17—O4—C22	119.4 (3)
C2—C1—C6	121.6 (7)	C28—O5—H5B	109.5
C2—C1—H1A	119.2	C20—C15—C16	116.9 (5)
C6—C1—H1A	119.2	C20—C15—H15A	121.6
C14—O2—H2B	109.5	C16—C15—H15A	121.6
C1—C2—C3	118.2 (6)	C17—C16—C15	123.5 (5)
C1—C2—H2A	120.9	C17—C16—H16A	118.3
C3—C2—H2A	120.9	C15—C16—H16A	118.3
C2—C3—C4	121.9 (5)	O4—C17—C18	118.3 (4)
C2—C3—O1	119.4 (5)	O4—C17—C16	124.6 (4)
C4—C3—O1	118.7 (5)	C18—C17—C16	117.1 (4)
C5—C4—C3	117.7 (6)	C17—C18—C19	121.7 (5)
C5—C4—Cl1	122.6 (5)	C17—C18—Cl2	118.5 (4)
C3—C4—Cl1	119.7 (4)	C19—C18—Cl2	119.8 (4)
C6—C5—C4	122.5 (6)	C20—C19—C18	119.6 (5)
C6—C5—H5A	118.7	C20—C19—H19A	120.2
C4—C5—H5A	118.7	C18—C19—H19A	120.2
C5—C6—C1	118.0 (7)	C19—C20—C15	121.3 (5)
C5—C6—C7	122.4 (8)	C19—C20—C21	120.7 (6)
C1—C6—C7	119.5 (8)	C15—C20—C21	118.0 (6)
F3—C7—F2	126.1 (8)	F6—C21—F5	108.5 (7)
F3—C7—F1	98.6 (7)	F6—C21—F4	106.3 (7)
F2—C7—F1	97.6 (7)	F5—C21—F4	91.8 (7)
F3—C7—C6	111.7 (7)	F6—C21—C20	121.0 (8)
F2—C7—C6	110.8 (8)	F5—C21—C20	117.1 (6)
F1—C7—C6	108.9 (7)	F4—C21—C20	107.6 (6)
O1—C8—C13	117.0 (4)	C23—C22—C27	123.6 (4)
O1—C8—C9	122.8 (4)	C23—C22—O4	119.6 (4)
C13—C8—C9	120.0 (4)	C27—C22—O4	116.6 (4)
C10—C9—C8	118.8 (5)	C22—C23—C24	118.1 (4)
C10—C9—H9A	120.6	C22—C23—H23A	121.0
C8—C9—H9A	120.6	C24—C23—H23A	121.0
C9—C10—C11	123.2 (5)	C25—C24—C23	119.8 (4)
C9—C10—H10A	118.4	C25—C24—H24A	120.1
C11—C10—H10A	118.4	C23—C24—H24A	120.1
C10—C11—C12	117.3 (5)	C24—C25—C26	120.8 (4)
C10—C11—H11A	121.4	C24—C25—H25A	119.6
C12—C11—H11A	121.4	C26—C25—H25A	119.6
C13—C12—C11	121.1 (4)	C25—C26—C27	120.5 (4)
C13—C12—C14	119.2 (4)	C25—C26—C28	120.2 (4)
C11—C12—C14	119.6 (4)	C27—C26—C28	119.4 (4)
C8—C13—C12	119.4 (5)	C22—C27—C26	117.2 (4)
C8—C13—H13A	120.3	C22—C27—H27A	121.4
C12—C13—H13A	120.3	C26—C27—H27A	121.4
O3—C14—O2	122.8 (4)	O6—C28—O5	121.4 (4)
O3—C14—C12	118.7 (4)	O6—C28—C26	120.8 (4)
O2—C14—C12	118.4 (4)	O5—C28—C26	117.8 (4)
			
C6—C1—C2—C3	4.0 (10)	C20—C15—C16—C17	1.4 (8)
C1—C2—C3—C4	−3.2 (9)	C22—O4—C17—C18	−157.8 (4)
C1—C2—C3—O1	178.3 (5)	C22—O4—C17—C16	23.2 (7)
C8—O1—C3—C2	−56.3 (7)	C15—C16—C17—O4	177.6 (5)
C8—O1—C3—C4	125.2 (5)	C15—C16—C17—C18	−1.5 (8)
C2—C3—C4—C5	1.8 (8)	O4—C17—C18—C19	−178.6 (5)
O1—C3—C4—C5	−179.7 (5)	C16—C17—C18—C19	0.5 (8)
C2—C3—C4—Cl1	179.9 (4)	O4—C17—C18—Cl2	2.3 (6)
O1—C3—C4—Cl1	−1.6 (7)	C16—C17—C18—Cl2	−178.6 (4)
C3—C4—C5—C6	−1.2 (9)	C17—C18—C19—C20	0.6 (8)
Cl1—C4—C5—C6	−179.2 (5)	Cl2—C18—C19—C20	179.6 (4)
C4—C5—C6—C1	2.0 (10)	C18—C19—C20—C15	−0.6 (8)
C4—C5—C6—C7	178.8 (6)	C18—C19—C20—C21	178.7 (6)
C2—C1—C6—C5	−3.5 (10)	C16—C15—C20—C19	−0.3 (8)
C2—C1—C6—C7	179.6 (7)	C16—C15—C20—C21	−179.7 (6)
C5—C6—C7—F3	−146.2 (7)	C19—C20—C21—F6	−52.4 (11)
C1—C6—C7—F3	30.6 (11)	C15—C20—C21—F6	127.0 (8)
C5—C6—C7—F2	−0.2 (11)	C19—C20—C21—F5	171.4 (7)
C1—C6—C7—F2	176.6 (6)	C15—C20—C21—F5	−9.2 (11)
C5—C6—C7—F1	106.0 (9)	C19—C20—C21—F4	69.9 (8)
C1—C6—C7—F1	−77.2 (9)	C15—C20—C21—F4	−110.7 (6)
C3—O1—C8—C13	143.9 (5)	C17—O4—C22—C23	55.1 (6)
C3—O1—C8—C9	−41.9 (7)	C17—O4—C22—C27	−129.0 (4)
O1—C8—C9—C10	−177.9 (5)	C27—C22—C23—C24	−0.3 (7)
C13—C8—C9—C10	−3.9 (8)	O4—C22—C23—C24	175.4 (4)
C8—C9—C10—C11	6.0 (8)	C22—C23—C24—C25	1.3 (7)
C9—C10—C11—C12	−5.3 (8)	C23—C24—C25—C26	−2.2 (7)
C10—C11—C12—C13	2.5 (7)	C24—C25—C26—C27	2.1 (7)
C10—C11—C12—C14	−178.8 (4)	C24—C25—C26—C28	−179.6 (4)
O1—C8—C13—C12	175.6 (4)	C23—C22—C27—C26	0.2 (7)
C9—C8—C13—C12	1.3 (7)	O4—C22—C27—C26	−175.6 (4)
C11—C12—C13—C8	−0.7 (7)	C25—C26—C27—C22	−1.0 (6)
C14—C12—C13—C8	−179.4 (4)	C28—C26—C27—C22	−179.3 (4)
C13—C12—C14—O3	178.2 (4)	C25—C26—C28—O6	−1.7 (7)
C11—C12—C14—O3	−0.5 (7)	C27—C26—C28—O6	176.6 (4)
C13—C12—C14—O2	−4.1 (7)	C25—C26—C28—O5	177.3 (4)
C11—C12—C14—O2	177.1 (4)	C27—C26—C28—O5	−4.4 (6)

### Hydrogen-bond geometry (Å, °)

**Table:**

*D*—H···*A*	*D*—H	H···*A*	*D*···*A*	*D*—H···*A*
O2—H2B···O6	0.82	1.79	2.599 (4)	168.
O5—H5B···O3	0.82	1.82	2.629 (4)	170.
